# The Application of Fiber-Reinforced Materials in Disc Repair

**DOI:** 10.1155/2013/714103

**Published:** 2013-12-08

**Authors:** Bao-Qing Pei, Hui Li, Gang Zhu, De-Yu Li, Yu-Bo Fan, Shu-Qin Wu

**Affiliations:** Key Laboratory for Biomechanics and Mechanobiology of Ministry of Education, School of Biological Science and Medical Engineering, Beihang University, Beijing 100191, China

## Abstract

The intervertebral disc degeneration and injury are the most common spinal diseases with tremendous financial and social implications. Regenerative therapies for disc repair are promising treatments. Fiber-reinforced materials (FRMs) are a kind of composites by embedding the fibers into the matrix materials. FRMs can maintain the original properties of the matrix and enhance the mechanical properties. By now, there are still some problems for disc repair such as the unsatisfied static strength and dynamic properties for disc implants. The application of FRMs may resolve these problems to some extent. In this review, six parts such as background of FRMs in tissue repair, the comparison of mechanical properties between natural disc and some typical FRMs, the repair standard and FRMs applications in disc repair, and the possible research directions for FRMs' in the future are stated.

## 1. Introduction

**Figure 1:**
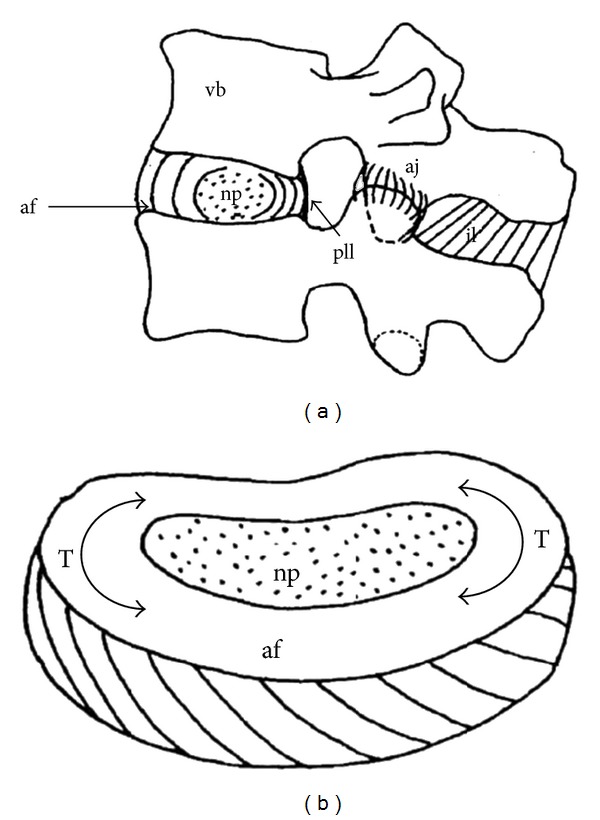
Schematic structure of the IVD and NP-AF interaction under compression [4]. vb: verterbral body; af: annulus fibrosus; np: nucleus pulposus; aj: apophyseal joint; pll: posterior longitudinal ligament; il: interspinous ligaments; T: tensile stress.

The intervertebral disc (IVD) is a heterogeneous, cartilaginous structure which contributes to the flexibility and load support in the spine. It consists of three parts: the nucleus pulposus (NP) in the center, the annulus fibrosus (AF) peripherally, and the cartilaginous endplates (CE) [1]. The fluid NP delivers loads to the AF in the form of swelling pressure while the multilayer and angle-ply AF wraps NP like a net bag guarded against the excessive expansion of NP (Figure 1). The direction of the fibers was angled varying from 40° to 70° to the vertical axis [1]. The intervertebral disc is in contact with the vertebral bodies through CE which is responsible for the exchange of substance through the microporous structure [2]. Anyone off normal of these three parts may cause the disc degeneration.

The spine disease which is related to the disc degeneration affects human health and normal life. The treatment and regeneration of the degenerated disc is one of the most urgent current clinical problems. There are complex mechanical and structures requirements for the disc repair. The fluid nature of the NP ensures the compressive loading applied to a disc to generate a tensile hoop stress (T) in the annulus (Figure 1) [3, 4]. Meanwhile, since the IVD joint possess 6-degree freedom of motion, AF needs to bear axial and radial compression and stretch as well [5]. According to the *in vivo* and *vitro* results, the interdiscal pressure under 300 N loading is approximately 1.3 MPa, the circumference stretch stress of the AF is 12.7 MPa [6], and the shear modulus is 25~110 KPa [7]. In this view, high mechanical properties and complex load bearing capacity are necessary for IVD repair materials.

Many methods for disc restoration are aimed at the reinforcement of disc mechanical properties, such as injection of the polymethylmethacrylate for the traditional intervertebral injury and fracture [8], the usage of biocompatible cage and metal internal fixations for bone fusion, and preventing the intervertebral collapse and failure restoration [9]. Some chemistry technologies such as doubly cross-linked microgels can make the strength enhanced by 3 to 5 times compared to original materials and dynamic properties similar to the health disc [10]. The fiber-reinforced technology is commonly used for increasing the mechanical properties of materials and some researchers are trying to use FRMs into the field of disc repair.

Many biocompatible and biodegradable fiber-reinforced polymers combined with matrix form new mechanical enhanced FRMs and have mature application in bone repair field. The calcium phosphate cement (CPC) reinforced with polycaprolactone polymer (PCL) fibers in different weight increased the modulus nearly twofold from 85 to 155 Mpa [16]. CPC power matrix addition to poly(coglycolide) (PLGA) fibers makes the compressive and bending strength close to the bone [17]. 15 wt% self-reinforced poly-L-lactide (PLLA) fiber coated with hydroxyapatite (HA) composite makes the original bending modulus increase from 8.3 Gpa to 9.7 Gpa [18]. Multi-fiber-reinforced CPC compound with the compressive strength from 0.9 to 69 Mpa [19] and the energy to fracture increase by 390X compared to pure CPC, with bending strength range from 1.2 to 60 Mpa. Chitosan fiber-reinforced composite which possesses well-rheological behavior [20] and biodegradability [21] repairs 40 mm goat shank bone defect successfully after 15 weeks, with the chitosan FRM group recovering to the same level BMD and bone mechanical strength to the intact group [22–26]. The porous three-dimensional poly(L-lactic acid) scaffold reinforced by the chitin fibers with link is an appropriate scaffold for tissue engineering [27]. As for the cell level, the material microstructure after fiber being reinforced is more suitable for cell adhesion and reproduction. Poly(3-caprolactone) (PCL)/b-tricalcium phosphate (TCP) nanofibre-reinforced hierarchical collagen scaffolds cell seeding efficiency increased from 55% for pure collagen scaffolds to 78% after fiber reinforced with tensile modulus increased by 7 times as well [28]. The carbon nanotube reinforced materials have a better ability to adsorb proteins and better cell attachment, proliferation, and differentiation than graphite [29], so they could also induced more bone formation [30]. Hydrogel is widely used for many soft tissues and is supposed to be the most promising disc repair materials [31]. However, pure hydrogel is proved to be insufficient for disc mechanical restoration [32]. Elastin fiber-reinforced hydrogel makes a better performance and can achieve cartilage-like properties [33]. The hyaluronic acid matrix reinforced by cellulose and elastin-like polypeptide fibers with adding collagen, hyaluronic acid, and chondroitin sulfate can obtain a better collision property [34–36]. Although these researches have not reached the stage of tissue fabrication or the *in vivo* and *vitro* experiments, the composites' properties got enhancement through the fiber-reinforced technology.

Scaffolds play fatal roles in both disc arthroplasty and whole disc replacement tissue regeneration [37–40]. The FRMs are becoming the ideal material for kinds of hard and soft tissue regeneration. A combination of matrix materials and different fibers arrays may help address the weakness of each component as a scaffold and supply easy grown mechanical and structure environments. So far, FRMs have been widely used in bone repair and bone tissue engineering [41]. A great many of fibers may be used for fiber-reinforced material fabrication such as polymer fibers such as polyethylene (PE), polylactic acid (PLA), and chitosan [42], biological ceramic fibers like bioglass, HA and calcium polyphosphate [43], and metallic fibers such as stainless steel and titanium [44].

This review addresses the mechanical requirement for disc tissue repair, compares the mechanical properties of FRM and disc tissue, and summarizes typical FRMs applications in disc tissue engineering. The adjustable mechanical properties by different spatial configuration of the fibers and their impacts on the cell growth have also been discussed preliminarily. Currently, the definition and summary about the FRMs for disc repair are not that much. The developments of FRMs in the field of disc tissue repair have very important practical applications and biomimetic meanings for the clinical spinal surgery.

## 2. Comparison of Mechanical Properties between Nature Disc and FRMs

### 2.1. The Mechanical Properties of Healthy Human Disc

The properties of the disc as a whole are determined by the mechanical behavior of the NP. NP is the most active element of the physiological hydrodynamical system of the intervertebral disc extradiscal space [56]. The most important characteristic of this system is the intradiscal pressure (IP) which depends on the external loads and the degree of hydration of the nucleus. The IP when being in latericumbent position is 0.3234 Mpa and 0.8428 Mpa when sitting. The standing IP is 60~80% sitting pressure. In young people aged from 20 to 30, the vertical stretch strength is about 0.196 Mpa, and the horizontal one is 0.294 Mpa. The vertical tensile strength of the outer layer AF is 15.68 Mpa and that of the inner layer is 6.664 Mpa. The horizontal outer layer is 7.84 Mpa and inner layer is 4.4 Mpa [45, 57]. The compressive stiffness is increased with loading while the modulus is not increased as significantly as the stiffness. The compressive stiffness and modulus are shown in Table 1. The *in vitro* human lumbar torsional stiffness is 2.0 Nm/deg [58], and the compressive stiffness is 2~14 KN [59]. The unconfined compressive elastic modulus of human disc is 5 kpa, and the Poisson ratio is 0.62, and relaxation rate is 65% [60]. The confined compressive elastic modulus is 0.14 Mpa, effective modulus is 1 Mpa, and permeability is 0.9*E* − 15 m4/Ns [61]. The torsional shear modulus is from 7.4 to 19.8 Kpa; phase angle is between 23° and 30° [60]. The disc system is a typical MKC oscillatory system, with inherent frequency about 4~5 Hz [62].

The NP mechanical properties are related to the content of glycosaminoglycan (GAG) and water. The normal NP swelling pressure is about 0.138 ± 0.029 MPa; effective aggregate modulus is 1.01 ± 0.43 MPa [63]. The average vertical strain is 2~8% and radial strain is 1~4% of human lumbar AF under neutral compression [64]. Healthy NP compressive strain is −10%~+10% [65]. The elastic modulus is 3~6 Kpa [66]. The single axial compression of the AF has obvious compliance, but after the strain increased to 20%~40%, the stiffness grows spurt. The horizontal shear compliance is smaller than the vertical ones. When the strain loads over 60%, the AF fracture not happened, but irreversible deformation happened [67].

### 2.2. Mechanical Properties of FRMs

The mechanical properties of FRMs increase with the content of fibers mostly, but the low density indicates a desired reinforcing storage and loss modulus of nanofibers [49]. It is possible to control the porosity and porous size through fiber-reinforced method, and the critical length of the fibers could be calculated using empirical formula, and so does the compressive strength of the scaffold using the fiber length [68]. Higher density fibers may result in a morphological change of the gel structure where the occurrence of nanofibers disrupts the continuity of the gel network and overall weakening of the construct. Meanwhile, the fiber processing technique may bring in bubble; the more the fibers added, the more the bubbles taken in, and this may be an important factor of modulus weaken ing.  Table 2 shows some FRMs for disc and disc-like joint and cartilage tissue engineering. The average compressive stiffness of the human lumbar when loaded by 577 ~ 2058 N was 1400 N/mm [69], while the biomimetic artificial IVD with fiber-reinforced annulus structure got a 2-fold stiffness of the normal disc [70]. Using 3D printed technique for the rat tissue engineered-total disc replacement (TE-TDR) with cell-seeded alginate and cell-seeded collage fibers was found a similar dynamic modulus (235 ± 51 kPa) with the native disc (238 ± 68 kPa) [71].

These FRMs are characterized by the following features. (1) Properties tunableness: a wide range of properties could be obtained through adjustment of fiber scales, processing technology, and geometry space configuration. (2) Good viscoelastic properties: body tissues are viscoelastic materials to some extent. The IVD restoration needs more significant viscosity properties than other tissues and load bearing strength as well. (3) Different fibers and matrix mix and match increase material diversity: as for this point, some researchers suggested that the focus should be on the deep boost of several certain mature compounds instead of keep searching for different materials with a smattering of knowledge of each one such as only culture cells for only days.

The main structure of the FRMs is the fiber. A study reported the diameters of the individual collage fibrils which are the main structural components of the NP and found that the nanoscale (with a mean diameter of 92.1 ± 26.54 nm) of the collage fibrils had a mild linear correlation with the compressive modulus of the NP [72]. The human fibrous tissue (FT) was compared with annulus and nucleus in relaxation and dynamic properties [60]. The percent relaxation of the FT was 90% included both AF and NP (70%~80%). The storage modulus of the fibrous tissue was also larger than that of the AF and NP. The FT is not proved to be a substitute for native tissue within the disc space.

Many researches regarded the constitutive equation of FRMs under different loading as very important research fields for FRMs tissue engineering. The models included anisotropic viscoelastic behavior under finite deformation [73, 74] and large-strain deformation [75] of soft composites. The stress transfer in collagen fibrils reinforcing tissues was infected by the fibril slenderness significantly. A large slenderness value led to high stress in a fibril and it is beneficially provided since they do not exceed the fracture stress of collagen. Fibers with taper-type shape are better than fibers with uniform cylindrical shape in against fiber fracture [76].

### 2.3. FRMs and Cell Culture

The small porosity of some high densely arranged nanofiber-reinforced materials makes it difficult for cell permeating into the fiber bundles and leads to abnormal ECM environment, so the material restoration fails to recover a normal tissue mechanical and physical standard finally. In response to this issue, a new method called dynamic cell culture technique can enhance the permeability of the stem cell and the quality of the ECMs [77]. NP cell bears the hydrostatic pressure while the cells between AF and NP enduring the deformation stimulate mostly. The cell geometry is impacted by the micromechanical environment which is affected by the fiber-reinforced technique, and the adapt law of the cell development direction is the reduction of the strain load [78].

The cell growth situation is affected by the material scale. Nanoscaled fibers which can stimulated a variety of interactions at the cellular level may promote greater amounts of specific protein interactions and more efficiently new bone formation [79]. The microstructured calcium phosphate materials concentrate more proteins and also are proved to induce more bone formation. The biocompatibility and bioactivity are also promoted by the nanoscaled materials [80]. The carbon nanotubes can induce cells in soft tissues to form inductive bone by concentrating more proteins including bone-inducing proteins [81, 82], which is also the contribution of the nanoscale structure. Though the nanoscale materials have stimulative effects on cell growth and induced differentiation, the biocompatibility and toxicity still need cautious experiments before *in vivo* attempt [83].

## 3. The Disc Restoration Objects

The intervertebral disc (IVD) is the mechanical and structural unit of the spine, so the functional restoration is very important and needs some standard indicators for evaluation. There are many mechanical characters of the IVD including nonlinearity, viscoelasticity, anisotropy, heterogeneity, and permeability. Finding limited crucial and reasonable properties is more meaningful. One of the clinical golden standards for IVD examination is the IVD height from MRI and X-ray plain film [84]. As for the experimental purpose, the neutral zone stiffness and the relative length during axial low load can estimate the recovery condition from needle damage and the endplate injury. As for large and severe AF defects and IVD degeneration, the torsion strength and ROM can test the restoration effects [84]. The dynamic modulus and stress relaxation and creep test make a judgment of the viscoelasticity behavior of the repaired disc [53]. For the implantable tissue replacement methods, shear strength of the interface is very crucial indicator of tissue fusion [85].

The synthesis and maintenance of extracellular matrix (ECM) are necessary for tissue activity and cell reproduction. The collage and GAGs are main substances for disc ECM [86]. Immunohistochemical methods can make qualitative and quantitative measurement for these two ingredients. The DNA transfer and mRNA expression can reflect the activity and propagation of newborn cells [87]. Though the cell-based therapy methods are more mature than whole IVD transplantation, the inadequacy for severe degeneration and disc prolapse stage limited its development and more and more focuses are on the latter. By now, whole IVD tissue engineering method remains under the exploration and experimentation step. Generally, it needs stages from a material to an artificial tissue use  *in vivo*. The duration selections for the stages are shown in Table 3. At present, the time span for each period has not obtained a unified time yet.

## 4. The Application of FRM in Disc Arthroplasty

As was mentioned in the front part, some materials such as coralline and hydroxyapatite could induce osteogenesis. Spinal fusion is widely used in clinical for intervertebral decompression which aims at complete bony fusion. So, these materials have good performance for intervertebral fusion, especially after being reinforced by fibers such as PLA, PEG, or carbon fibers [88–90]. Though fusion is not the ideal choice for disc repair, FRMs used for fusion materials are also a research hotspot at present.

The materials for disc replacement prosthesis need to possess different mechanical properties and composite structures. Flexibility, toughness, and high strength are basic characteristics of the soft biological tissues. For this reason, by employing materials with a single structural arrangement, it is not possible to combine all of these features. So, the structure of natural disc has been reproduced by adopting a biomimetic approach. This led to the development of a fiber-reinforced hydrogel able to match the performances of the natural disc and those of the surrounding tissues [91].

Disc arthroplasty is a kind of disc therapy which uses disc prosthesis (DP) with no biological activities for disc replacement and functional disc repair. The metallic and high polymer-core DP incurs DP sinking and wear and also ADD (adjacent disc degeneration) because of the high stiffness of the DP materials. The ideal artificial disc should be biomimetic which means that the mechanical properties, structure constitution and the motion function are similar with the nature disc tissue. Since, 1990s researchers have begun to use polyethylene and polyurethane fiber-reinforced silicon elastomers to make disc spacers. The artificial disc obtained the same compressive and torsional properties through adjusting the fiber direction, numbers of fiber layers, and the sequence of reinforcement [92]. The compressive modulus increased with the fiber angle and fiber content positively [93].

Hydrogel is always used for disc tissue engineering materials for its close properties to IVD tissue. Using fibers to reinforce hydrogel makes the composite with better stiffness and strength and maintenance of original properties. After fiber have added, the water absorption rate decreased for the new FRMs, is 30 wt% fibers addition with only 25% water absorption [93]. This rate is the same as the collage in disc, and the FRM with 30 wt% fibers could achieve suitable mechanical properties demanded by the normal IVD.

**Figure 2:**
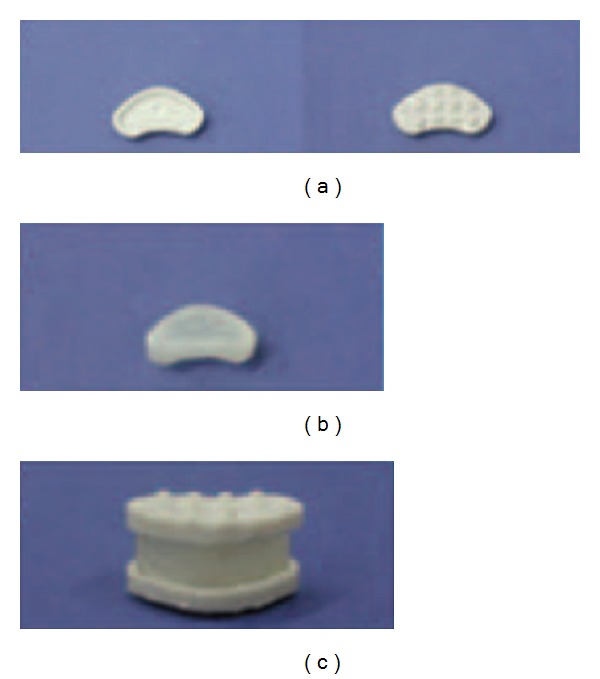
The parts and the whole of the disc prosthesis. (a) Two sides of HAPEX endplates, (b) composite hydrogel for IVD substitute, and (c) total IVD substitute prototype [11].

Gloria et al. [11] developed a new disc prosthesis using poly(2-hydroxyethyl methacrylate)/poly(methyl methacrylate) (PHEMA/PMMA) (80/20 w/w) semi-interpenetrating polymer network (s-IPN) composite hydrogel reinforced with poly(ethylene terephthalate) (PET) fibers as annulus/nucleus substitute and two hydroxyapatite-reinforced polyethylene composites (HAPEXTM) as endplates as was shown in Figure 2. This FRMs' disc performed enough static properties compared to normal disc, with maximum static compressive stiffness 4030 ± 612 N/mm, torsional rigidity 2.8 ± 0.3 Nm/deg, and shear stiffness 205 ± 22.1 N/mm. The ten million times fatigue test indicated that no damage or wear happened during the test, which is much better than some products in the markets. Meanwhile, the hydrogel matrix FRMs' disc has the similar dynamic and viscoelastic properties for the healthy disc to the other prosthesis.

## 5. The Application of FRM in Disc Tissue Engineering

It is more and more prevalent for disc restoration using tissue engineering method. The cell-based method includes two main directions which are the cell transplantation and the bioactive tissue transplantation. The bioactive tissue material used for disc repair is a research focus at present. The microstructure of the FRMs [94] is proved to promote cell adhesion and growth by supporting a 3D growing space [95]. Some studies have used FRMs for disc repair and various fiber features may lead to different repair effects.

The main cells used for disc regeneration are NP cell, AF cell, and stem cells. The NP cells and AF cells are like the chondrocyte and fibroblast, respectively, in both structure and function. These two kinds of cells are always used for testing materials bioactivity. Some biocompatibility (BC) matrixes after reinforced by the biodegradable nanofibers may improve the brittleness of single fibers and the fiber density become less dense after reinforcing the matrix, making cells easier grow into the fibers. The fibroblasts were cultured in single array and square crossing fibers in FRMs, respectively, and were found to grow along the fibers as a line in the single array group and distribute in the meshes in the crossing one. The nanoscale FRMs process abilities to induce cell grow directions [96].

**Figure 3:**
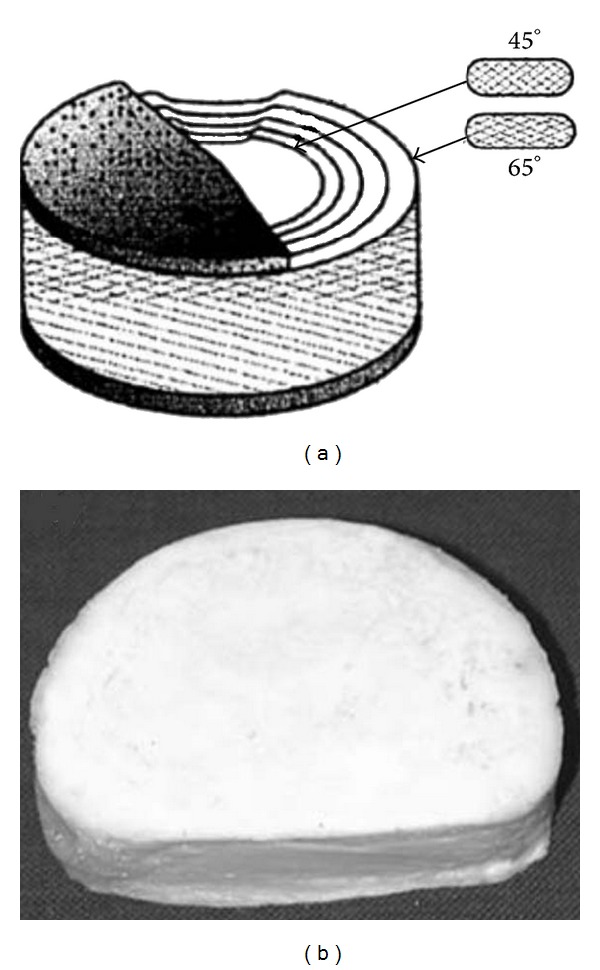
(a) A schematic representation of the fiber-reinforced disc substitute with hydroxyapatite reinforcing hydrogel endplates, (b) The total intervertebral disc substitute prototype [12].

The mechanical strength of the composite may be enhanced after fiber being reinforced. We could obtain certain properties through increasing fiber content and changing fiber angles. PET fibers' angle changeing from 45° to 60° (shown in Figure 3) (angle to the load) which were added into hydrogel makes compressive modulus (CM) increased by 2~3 times. After changing volume fraction of PCL fibers from 0% to 30%, CM increases by 15 times [12]. PGA (polyglycolic acid) is reinforced by PLA fibers; CM increased with volume of PLA fibers linearly, which change by 0%~68% CM increased by 20 times [97]. 3%~17% volume change of elastic fibers was reinforced by collage fibers; the stretch modulus increased by 1 time with the same fiber angles while the angle change for 15 not significant changed the stretch modulus.

Researchers used PCL (polycaprolactone) nanofiber-reinforced hydrogel for NP tissue engineering. The dynamic properties of the materials are not increased with fiber density; on the contrary lower density obtained a closer storage and loss modulus to the real disc. We may draw a conclusion that fiber content got a bigger weighting factor than the fiber angles on the impact of mechanical properties. Change angles may adjust FRMs in a small range.

**Figure 4:**
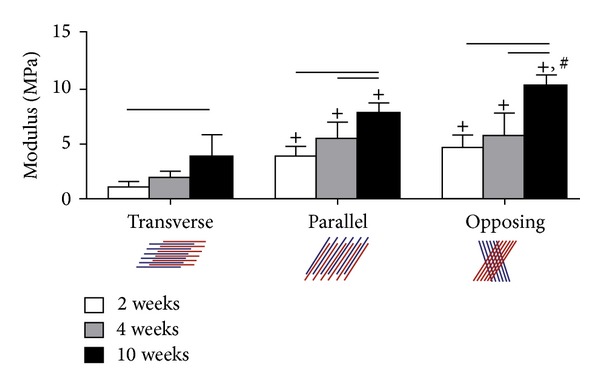
Three type of fiber organization between layers and the shear modulus when loaded tensile [13].

The annulus fibrosus is a natural fiber-reinforced structured tissue. Some researches focus on the organization of collagen fibers into planes of alternating alignment and found that it played an important role in annulus fibrosus tissue function. By using MSC-seeded nanofibrous scaffolds and applying the constitutive model to uniaxial tensile stress-strain data for bilayers with three different fiber orientations, they found that fiber orientation of adjacent layers with an opposing style got the biggest strength against the shear between layers under tensile load as shown in Figure 4 [13].

## 6. Conclusion and Perspectives

Current therapies for disc degeneration and spinal disease mainly focus on the relieving pains instantly instead of functional and physiological repair on the long run. Tissue engineering provides new treatment strategy for disc repair. The application of fiber-reinforced materials in tissue repair has a wide range of use and mature background, considering the excellent mechanical properties, and will make a new direction for disc restoration.

FRMs are on the developing stage for disc repair; some problems and research fields such as the choice of fibers, the interaction of fibers-matrix, and also the processing technology effects for properties coordination deserve more deeply discussion. Meanwhile, the mechanisms of FRMs for cell growth and propagation are still not known yet.

MSC differentiation *in vivo* technique is widely studied for tissue regeneration. Recent research shows that the MSCs' function is related to the substrate stiffness which could be adjusted by fiber-reinforced methods [98, 99]. As the disc cell lives in hypoxia, it has been proved that MSC differentiation shows better cell survival rate and activity under low oxygen environment [100]. By controlling technology and fiber density, FRMs could obtain nanoscale 3D cell growth space and fulfill the oxygen concentration control.

**Figure 5:**
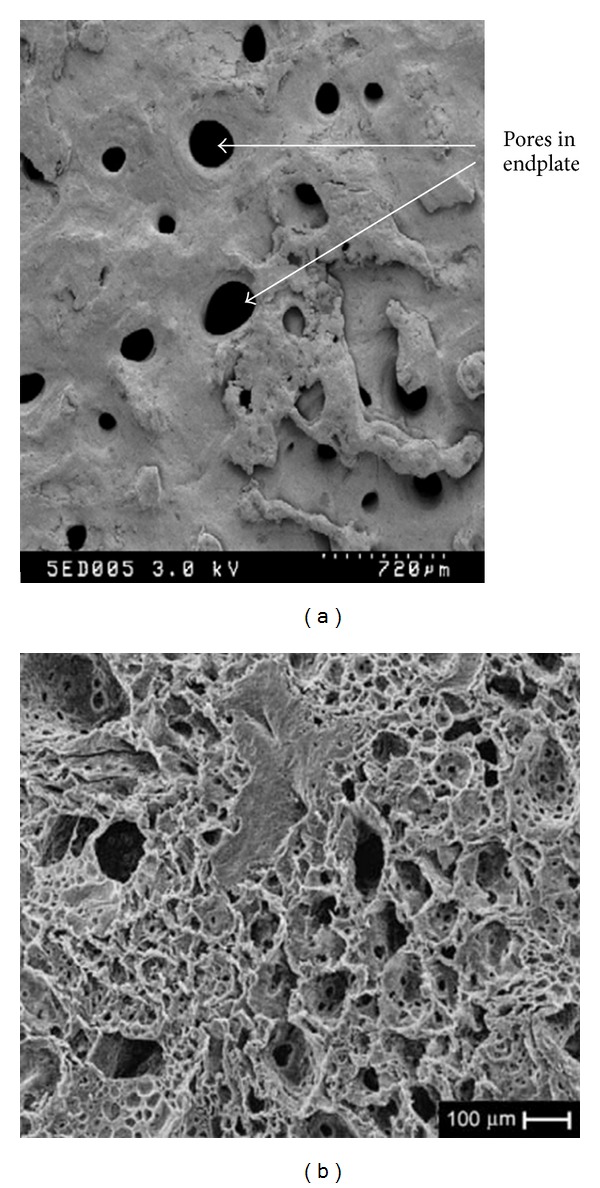
The similar scale of the pores in endplate and pores in FRM composite. (a) REM image of a vertebral endplate with 720 *μ*m scale plate [14] and (b) SEM analysis of FRM scaffolds with a 100 *μ*m scale plate [15].

The cartilage endplates of the disc have very important structure and many disc diseases originate form endplate degeneration. As for the special position, the junction of the vertebra, and disc tissue, the endplates are responsible for material exchange. The pores in the endplate (shown in Figure 5(a)) make it possible for mass metabolism between the inside and outside [14]. So the repair of this structure needs not only mechanical function but also diffusion and interface fusion properties. FRMs have a better bone fusion ability and can show better interface strength. At the same time, the microporous (shown in Figure 5(b)) formed by the embedded fibers may ensure the diffusion function [15]. Also, the endplates' tissue replacement needs two different properties in one piece of materials. FRMs' coordinability properties make this possible for a functional endplate.

## Figures and Tables

**Table 1:** Compressive stiffness and modulus of healthy human discs.

Load (N)	Stiffness (N/mm)	Modulus (Mpa) [45]
490	392–490	16.366
490–1470	666.4–3479	8.624~26.166
1470–9800	1400–7791	9.604~21.364

**Table 2:** Typical FRM use in disc implants and some similar tissues.

Fibers	Matrix	Mechanical properties			Fiber scale	Processing	Repaired tissue	literature
		Breaking stress (Mpa)	Breaking strain (%)	Elastic modulus (Mpa)				
Elastin	Hydrogel	0.08~2.08	10.6~247	0.8~3.68	Mircon	3D syringe drops crossed ‘‘log-piles”	Cartilage	Agrawal et al., 2013 [33]
PCL	Gelatin				Nano	Electrospun	AF	Beachley and Wen, 2009 [46]
	Dry	5~25		20~120				
	Wet	0.1~0.9		1~10				
Collagen	Elastin-like	1.85~4.08	23~314	5.3~33.1	Mircon	Winding	Abdominal wall	Caves et al., 2011 [47]
Polydioxanone (PDO)	PLA					Electrospun	AF	Cont et al., 2013 [48]
PCL	Hydrogel	Storage modulus	Loss modulus		Nano	Electrospun	NP	Thorvaldsson et al., 2012 [49]
		0.03 Mpa	0.006 Mpa					
N-vinyl-2-pyrrol-idone (NFC)	Hydrogel	Storage modulus	Loss modulus	0.02~8		Curing	NP	Borges et al., 2010 [50]
		0.14 Mpa	0.019 Mpa					

**Table 3:** Time span for experimental validation.

Experimental objects	Plant mode	Culture *ex vivo* (week)	Culture *in vivo* (month)
Canine model [51]	Cell-based		3, 6, 9, 12
Polymer scaffolds [52]	Explant	4, 8, 12	
Rodent model [53]	Implant		6
Collagen-gel compound [54]	Explant	3 days	
Alginate composite [55]	Explant	4	
